# Long-term inorganic nitrate administration protects against myocardial ischemia-reperfusion injury in female rats

**DOI:** 10.1186/s12872-023-03425-2

**Published:** 2023-08-21

**Authors:** Younes Yassaghi, Sajad Jeddi, Nasibeh Yousefzadeh, Khosrow Kashfi, Asghar Ghasemi

**Affiliations:** 1 Endocrine Physiology Research Center, Research Institute for Endocrine Sciences, Shahid Beheshti University of Medical Sciences, No. 24, Parvaneh Street, Yaman Street, P.O. Box: 19395-4763, Velenjak, Tehran Iran; 2 Department of Molecular, Cellular, and Biomedical Sciences, Sophie Davis School of Biomedical Education, City University of New York School of Medicine, New York, USA

**Keywords:** Nitrate, Nitric oxide, Female rats, Myocardial ischemia-reperfusion injury, Inducible nitric oxide synthase, Endothelial nitric oxide synthase

## Abstract

**Background:**

The favorable effects of nitrate against myocardial ischemia-reperfusion injury (MIRI) have primarily focused on male rats and in short term. Here we determine the impact of long-term nitrate intervention on baseline cardiac function and the resistance to MIRI in female rats.

**Methods:**

Female Wistar rats were randomly divided into untreated and nitrate-treated (100 mg/L sodium nitrate in drinking water for 9 months) groups (n = 14/group). At intervention end, levels of serum progesterone, nitric oxide metabolites (NOx), heart NOx concentration, and mRNA expressions of NO synthase isoforms (NOS), i.e., endothelial (eNOS), neuronal (nNOS), and inducible (iNOS), were measured. Isolated hearts were exposed to ischemia, and cardiac function indices (CFI) recorded. When the ischemia-reperfusion (IR) period ended, infarct size, NO metabolites, eNOS, nNOS, and iNOS expression were measured.

**Results:**

Nitrate-treated rats had higher serum progesterone (29.8%, P = 0.013), NOx (31.6%, P = 0.035), and higher heart NOx (60.2%, P = 0.067), nitrite (131%, P = 0.018), and eNOS expression (200%, P = 0.005). Nitrate had no significant effects on baseline CFI but it increased recovery of left ventricular developed pressure (LVDP, 19%, P = 0.020), peak rate of positive (+ dp/dt, 16%, P = 0.006) and negative (–dp/dt, 14%, P = 0.014) changes in left ventricular pressure and decreased left ventricular end-diastolic pressure (LVEDP, 17%, P < 0.001) and infarct size (34%, P < 0.001). After the IR, the two groups had significantly different heart nitrite, nitrate, NOx, and eNOS and iNOS mRNA expressions.

**Conclusions:**

Long-term nitrate intervention increased the resistance to MIRI in female rats; this was associated with increased heart eNOS expression and circulating progesterone before ischemia and blunting ischemia-induced increased iNOS and decreased eNOS after MIRI.

**Supplementary Information:**

The online version contains supplementary material available at 10.1186/s12872-023-03425-2.

## Background

Ischemic heart disease (IHD) is the principal cause of death globally, with about 9 million related deaths registered in 2019 [1, 2]. Global IHD costs are estimated to surpass 1 trillion dollars by 2030 [3]. IHD frequently happens following the narrowing/blockage of the coronary arteries, and reperfusion reduces myocardial injury; however, reperfusion causes further damage, named myocardial ischemia-reperfusion injury (MIRI) [4]. Ischemic pre-conditioning or post-conditioning protects against MIRI in animals [5, 6]. However, these findings have not yet been effectively translated into humans [7]. Using conditioning agents that are more clinically feasible is another approach to reduce MIRI [8]. Nitrate and nitrite, which produce nitric oxide (NO), have been suggested as nutrition-based interventions for decreasing MIRI [9].

Decreased NO bioavailability contributes to increased MIRI [10]. NO in the heart is produced by both NO synthase (NOS)-dependent [via endothelial (eNOS), neuronal (nNOS), and inducible (iNOS) NOSs] and NOS-independent (nitrate-nitrite-NO) pathways [11]. Reduced eNOS and nNOS expression [9, 12, 13] exacerbates, whereas the eNOS overexpression [14, 15] decreases MIRI in male mice. In addition, pharmacological pre-conditioning with NO donors reduced infarct size and increased recovery of post-ischemic myocardial function in humans [16] and animals [17]. Therefore, it has been suggested that nitrite should be referred to as a dietary mineral to emphasize its natural occurrence [18] and that our food supply should be fortified with nitrate and nitrite [19]. Estimates indicate that dietary intervention with nitrate and nitrite can potentially decrease the risk of IHD by 21% and avoid 6 million deaths per year [18]. In support, consuming a vegetable-rich diet as a source of inorganic nitrate reduces the IHD risk and its mortality [20–23]. A meta-analysis of prospective cohorts (average follow-up of 10.68 years) showed a reduced risk of IHD mortality in subjects on a vegetarian diet compared to a non-vegetarian diet in both sexes [20]. In addition, a 4% reduced risk of coronary heart disease was observed with increasing one fruit/vegetable serving per day after 14 years and 8 years of follow-up in women and men, respectively [22].

Positive impacts of nitrate and nitrite against MIRI have primarily been investigated in the short term; 7 days of intervention by nitrite [24, 25] and nitrate [25, 26] decreases sensitivity to MIRI in mice. Only one study from our laboratory addressed the positive impact of nitrate against MIRI in isolated hearts for a relatively longer duration (60 days) in rats [9]. Whether outcomes in short-term experiments can be extrapolated to long-term periods remains to be determined. The other point is that most studies on the protective effects of nitrite/nitrate against MIRI in isolated hearts have been conducted in males even though NO metabolism shows sex-dependency; compared to men, women have increased activity of the nitrate-nitrite-NO pathway [27], oral nitrate-reducing capacity [28], whole-body NO production [29], eNOS expression [30], and eNOS-related cardioprotection after ischemia-reperfusion (IR) [31] and thus have lower MIRI [32]. Therefore, the current study was designed to investigate whether the long-term (9 months) nitrate intervention provides resistance against MIRI in female rats.

## Methods

### Ethics approval

All experiments of the current study were affirmed by the published guideline of the care and use of laboratory animals in Iran [33]. All experiments of the current study were also reported following ARRIVE guidelines [34]. The ethics committee of the Research Institute for Endocrine Sciences affiliated with the Shahid Beheshti University of Medical Sciences confirmed and approved all experimental procedures of the current study (Ethic Code: IR.SBMU.ENDOCRINE.REC.1400.105; Approved Date: 2021-12-07). The research question, study design features, and analysis plan were prepared before the study was started. The humane end point for this study was when the treatment period was ended.

### Study protocol

**Fig. 1:**
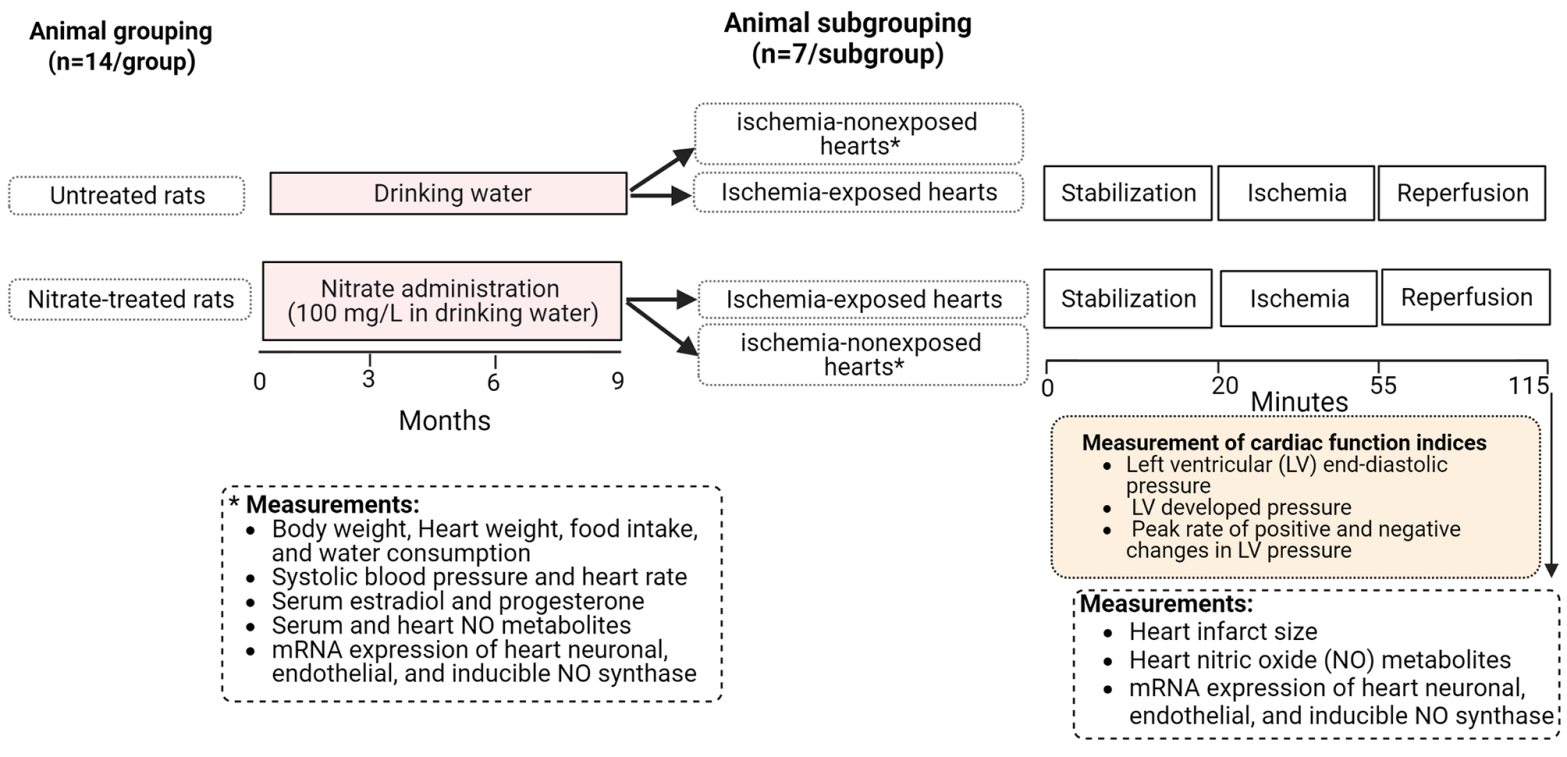
Experimental design of the study. Created with Biorender.com

This study is an experimental interventional study conducted in healthy female Wistar rats (8-month-old, 210–220 g) that were maintained in standard conditions with free access to a regular diet and drinking water. The study’s protocol is presented in Fig. 1. No rats were excluded from the beginning or during the study. Female rats (n = 28) were randomly allocated into 2 groups (n = 14/group), untreated and nitrate-treated groups, that consumed tap water and sodium nitrate in tap water (100 mg/L for 9 months), respectively. At the end of the nitrate intervention, each group was further allocated into 2 subgroups (n = 7/subgroup), of which hearts from one subgroup received IR (ischemia-exposed hearts) and the other did not (ischemia-nonexposed hearts). No adverse events were observed during the experiments.

After nitrate intervention, body weight (BW), heart weight (HW), food intake, and water consumption were measured, and the HW/BW ratio was calculated. Systolic blood pressure (SBP) was measured using the tail-cuff method, and heart rate (HR, by AD Instruments, MLT125R, Australia) was also measured. Blood samples were taken from the tail tips of nitrate-treated and untreated rats under isoflurane inhalation anesthesia, centrifuged (3000 g for 10 min), and sera conserved at -80 °C for subsequent quantifications of estradiol, progesterone, and NOx. In addition, after anesthetizing the rats with an intraperitoneal injection of sodium pentobarbital (60 mg/kg), their hearts were isolated, and the left ventricles (LV) of isolated hearts were kept at -80 °C for quantification of nitrite and nitrate, and expressions of eNOS, nNOS, and iNOS. To ensure adequate deep anesthesia, the tail and pedal of anesthetized rats were pinched until withdrawal reflexes were lost.

In another set of experiments, following stabilization for 20 min, isolated hearts from rats in untreated and nitrate-treated groups were subjected to a 35 min global ischemia and were then reperfused for 60 min. Cardiac function indices (CFI) were recorded throughout the experiment. Subsequent to the IR period, the hearts were isolated from the Langendorff apparatus and kept at -80 °C to measure infarct size, levels of NOx, and mRNA expression of eNOS, nNOS, and iNOS.

### Sample size calculation, randomization, and blindness of experimenters

According to our pervious study [9], evaluating the effect of nitrate administration on the tolerance to MIRI as a primary outcome, we determined that the standard deviations (S_1_ and S_2_) of left ventricular developed pressure (LVDP) in the control and nitrate-treated rats were 7.54 and 8.32, respectively, with the effect size being considered 20%. Using the formula below, we set a 2-sided α of 0.05 with a power of 80%, and calculated the sample size in each group to be 7.

$$n=\frac{({{z}_{1-{\upalpha }/2}+{z}_{1-\beta })}^{2}{{ \times (s}_{1}}^{2}+{{s}_{2}}^{2})}{{d}^{2}} = \frac{{(1.96+0.84)}^{2 }\times ({7.54}^{2}+{8.32}^{2})}{{11}^{2}} \cong$$ 7

In the current study, randomization was done using the Excel software’s random function, version 13.0 [35] (Additional File Fig. 1). Blinding was done at the outcome assessment levels, where the people who conducted the measurements were blinded to the experiment. To minimize potential confounders, sex hormone and NO metabolites (nitrite + nitrate, NOx) levels were measured simultaneously. Moreover, mRNA expression was measured in all groups in the presence of target and reference genes.

### Water consumption and food intake measurements

Rats were housed 3 per cage, and water bottles were filled with 500 mL of tap water and every three days, the remaining amount of water was measured. From this, each rat’s water consumption per day was calculated. Food intake was also measured similarly, starting with 300 g per cage.

### Assessment of serum sex hormones

Serum estradiol and progesterone concentrations were quantified by ELISA kits (Diagnostics Biochem, Ontario, Canada). Intra-assay coefficients of variation (CVs) and sensitivities were 5.2% and 10 pg/mL as well as 5.7% and 0.1 ng/mL for estradiol and progesterone, respectively.

### Assessment of SBP and HR

After the nitrate intervention, rats were placed separately in a restrainer at room temperature. The noninvasive tail-cuff method (AD Instruments, MLT125R, Australia) was used to measure in vivo SBP and HR. For each rat, SBP and HR values were the means of three consecutive recordings.

### Isolated heart and assessment of CFI

Nine months after nitrate intervention, rats were anesthetized with an intraperitoneal injection of sodium pentobarbital (60 mg/kg ), and their hearts were isolated by making an incision in the xiphoid-sternum and continuing to the lateral ends of the right and left costal margins. This incision then proceeded through the ribs at the right and left anterior axillary lines, creating a V-shaped thoracotomy. The heart was removed by transecting the descending aorta and inferior vena cava, followed by the ascending aorta and superior vena cava. The isolated heart was immediately transferred into ice-cold Krebs-Henseleit solution (composition in mM: 118.6 NaCl; 4.7 KCl; 2.5 CaCl_2_; 1.6 MgSO_4_; 1.2 KH_2_PO_4_; 25 NaHCO_3_; 11.1 glucose - all from Merck, Darmstadt, Germany), equilibrated with 95% O_2_:5% CO_2_, to minimize ischemic and hypoxic damage. The heart was then directly cannulated into the Langendorff apparatus, with perfusate dripping to avoid air embolism and microvascular obstruction. In the Langendorff system, the heart was retrogradely perfused with Krebs-Henseleit solution, causing the aortic valve to close. A deflated balloon was then inserted in the LV via the left atrium, inflated to 5–10 mm Hg, and connected via a water-filled polyethylene tube (PE-50) to a pressure transducer (MLT844-Sweden). The transducer was connected to a PowerLab system (AD Instruments, ML866, Australia) to record the CFI during stabilization (20 min), global ischemia (35 min), and reperfusion (60 min) periods. In this study, CFI include left ventricular end-diastolic pressure (LVEDP), LVDP, peak rate of positive (+ dp/dt), and negative (-dp/dt) changes in left ventricular pressure. Post-ischemic recovery of LVEDP, LVDP, and ± dp/dt were expressed as a percentage of initial values. The Krebs–Henseleit solution was maintained at 37 ± 0.5 °C and pH 7.4 throughout the experiment to ensure consistent physiological conditions for the isolated heart. The temperature (37 ± 0.5 °C) and humidity of the environment surrounding the heart were kept constant by a glass chamber. After the IR period, the hearts were isolated from the Langendorff apparatus and kept at -80 °C for future examinations.

### Assessment of serum and heart NO metabolites

At the end of nitrate intervention, 100 mg of heart tissues were homogenized in 500 µL phosphate-buffered saline and centrifuged (10 min at 10,000 g); NOx in all samples (serum and heart homogenates) were then quantified by the modified Griess method [36]. To deproteinize samples, zinc sulfate (10 µL, 15 mg/mL) [37] and NaOH (10 µL, 3.72 M) [38] were added to each sample, centrifuged at 10,000 g for 10 min, and supernatants were used for quantification of NOx. To quantify NOx concentrations, nitrate was converted to nitrite by adding 100 µL of vanadium trichloride (8 mg/mL in 1 M HCl), after which 50 µL N-(1-naphthyl) ethylenediamine (0.1% in ddH_2_O) and 50 µL of sulfanilamide (2% in 5% HCl) were added to the samples. Samples were then incubated for 30 min at 37 °C, and the optical density was read at 540 nm. Nitrite was quantified likewise, except that 1 M HCl was added to the samples to replace for vanadium trichloride. The nitrate concentration in heart samples was determined by subtracting nitrite from NOx concentrations. The Bradford method was used to measure protein concentration in the samples, and NOx levels are reported as per mg protein [39]. Intra-assay CVs of NOx in serum and heart tissue and nitrite in heart tissue were 2.4%, 2.9%, and 3.1%, respectively.

### Assessment of infarct size

After the IR period, the triphenyltetrazolium chloride (TTC) method was used to determine the infarct size in isolated hearts. In brief, the frosted, isolated hearts were sliced thinly, incubated in TTC (1% in phosphate buffer solution, 37 °C for 10 min), and embedded in formalin (10% for 24 h) to detect viable (red stained) from the necrotic (gray stained) areas. The sections were photographed, analyzed by Image J software, and represented as a percentage of the total area.

### Assessment of mRNA expression

The sequence of primers (eNOS, nNOS, iNOS, and ß-actin, as reference gene) is presented in Table 1. The RNX-Plus solution kit (Cinagen Co., Tehran, Iran) was used to extract RNA from LV of heart tissues. A cDNA synthesis kit (SMOBiO Technology, Taiwan) was used for cDNA synthesis. Ampliqon SYBR Green Master Mix (Ampliqon Company, Denmark) was used to amplify 1 µL of cDNA in a real-time PCR machine (Rotor-Gene 6000, Corbett, Life science, Australia).

**Table 1:** Sequences of primers for target genes

Name	Gene bank Accession No.	Sequence (5´→3´)	Size of product (bp)
Neuronal NOS	NM_052799	F: AATCTCAGGTCGGCCATCAC R: ATCCCCCAAGGTAGAGCCAT	126
Endothelial NOS	NM_021838.2	F: TGACCCTCACCGATACAACA R: CGGGTGTCTAGATCCATGC	100
Inducible NOS	NM_012611	F: TGGCCTCCCTCTGGAAAGA R: GGTGGTCCATGATGGTCACAT	93
ß-actin	NM_031144.3	F: CGTCCACCTGCTAGTACAAC R: CGACGACTAGCTCAGCGATA	100

NOS, nitric oxide synthase.

### Statistical analyses

Data analysis was performed by the GraphPad Prism software (Version 8), and is represented as mean ± SEM except for mRNA expressions, which are represented as relative fold changes. The Shapiro-Wilk test was used to assess the normal distribution of data except for mRNA expressions. All data showed a normal distribution and parametric statistical tests were used to analyze them. The Student’s t-test was used to compare the HW/BW ratio, SBP, HR, serum estradiol and progesterone concentrations, serum and heart NOx, CFI in untreated and nitrate-treated rats, and infarct size in ischemia-exposed and ischemia-nonexposed hearts. To compare food intake and water consumption, as well as CFI in ischemia-exposed and ischemia-nonexposed hearts at different time points, two-way mixed (between-within) ANOVA was applied, followed by the Bonferroni post-hoc test. Relative expressions of genes were calculated based on their cycle thresholds versus β-actin as a reference gene using the REST software, which uses a randomization test to compare the difference between samples [40]. The number of randomizations was set at 2000, which provides a reliable estimate of the P-value < 0.05 [40]. Two-sided P-values < 0.05 were considered statistically significant.

## Results

### Effects of nitrate intervention

Compared to the untreated group, the nitrate-treated group had lower BW (229.4 ± 3.9 vs. 248.7 ± 4.5 g, P = 0.007) but comparable food intake (16.4 ± 0.5 vs. 17.0 ± 0.4 g/day/rat), water consumption (26.6 ± 0.7 vs. 25.8 ± 0.8 mL/day), HW (1.25 ± 0.06 vs. 1.22 ± 0.05 g), and HW/BW ratio (0.55 ± 0.03 vs. 0.49 ± 0.03), as measured at the end of nitrate intervention. Nitrate also had no impact on SBP (97.7 ± 1.6 vs. 101.3 ± 1.4 mm Hg) and HR (317 ± 5 vs. 325 ± 7 beat/min).

Compared to the untreated group, the nitrate-treated group had higher serum progesterone (58.8 ± 1.4 vs. 45.7 ± 4.3 ng/mL, P = 0.013) but comparable serum estradiol concentration (52.7 ± 4.8 vs. 57.8 ± 10.9 pg/mL).

**Fig. 2:**
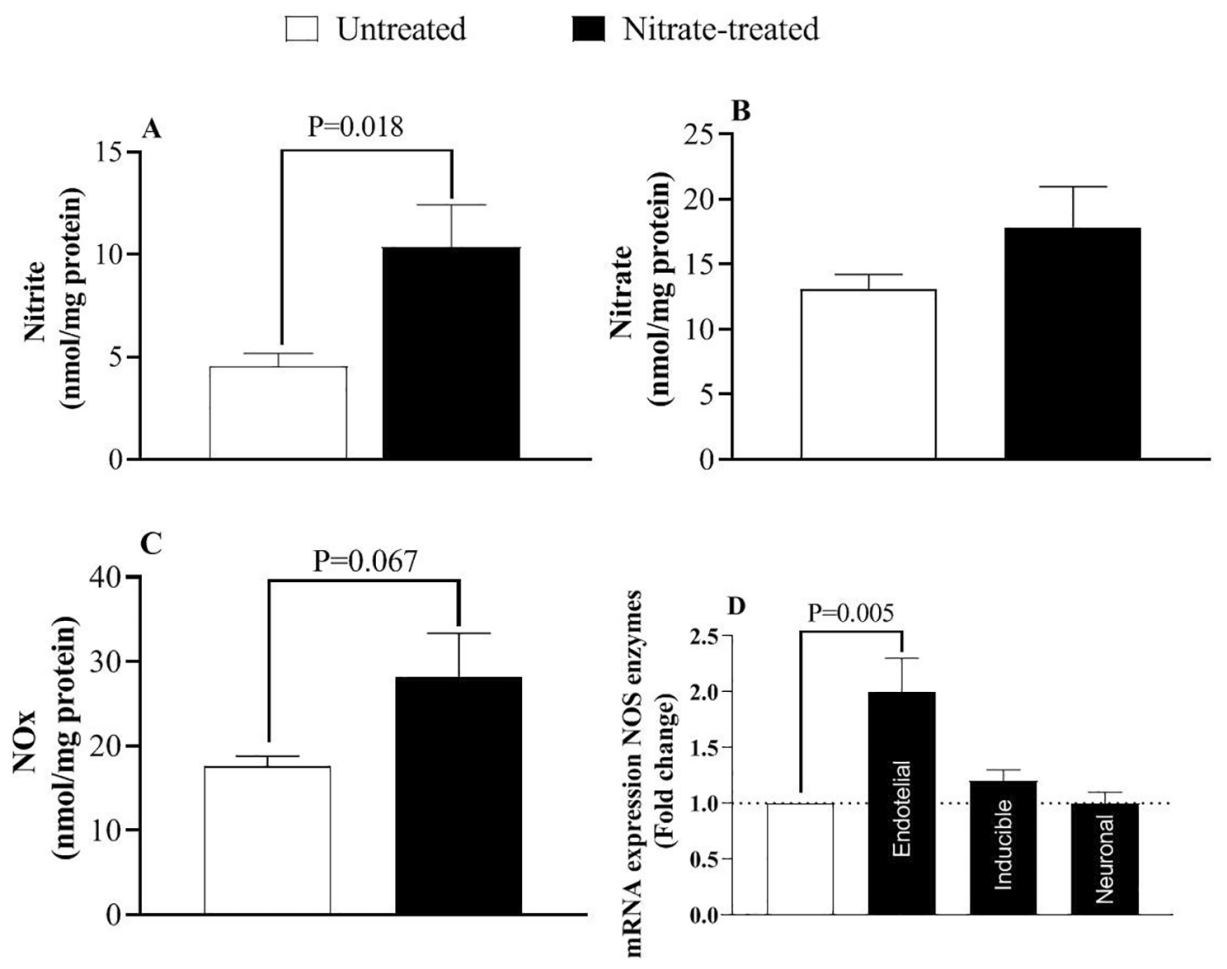
Nitric oxide metabolites (NOx, **A**, **B**, and **C**) and mRNA expression of NO synthase (NOS) enzymes **(D)** in hearts of rats following nitrate administration. (n = 7/subgroup)

Nitrate increased serum NOx levels by 31.6% (36.6 ± 3.0 vs. 27.8 ± 2.2 µmol/L, P = 0.035), indicating efficacy of the intervention. Moreover, nitrate increased heart nitrite and NOx by 131% (P = 0.018) and 60.2% (P = 0.067), respectively and eNOS expression by 2-fold (P = 0.005) (Fig. 2).

Finally, nitrate had no significant effects on CFI in the isolated hearts after 9 months, including LVDP (90.9 ± 2.8 vs. 92.7 ± 2.5 mm Hg), +dp/dt (2785 ± 129 vs. 2971 ± 73 mm Hg/s), and –dp/dt (2169 ± 81 vs. 2220 ± 114 mm Hg/s).

### Sensitivity to myocardial IR injury

**Fig. 3:**
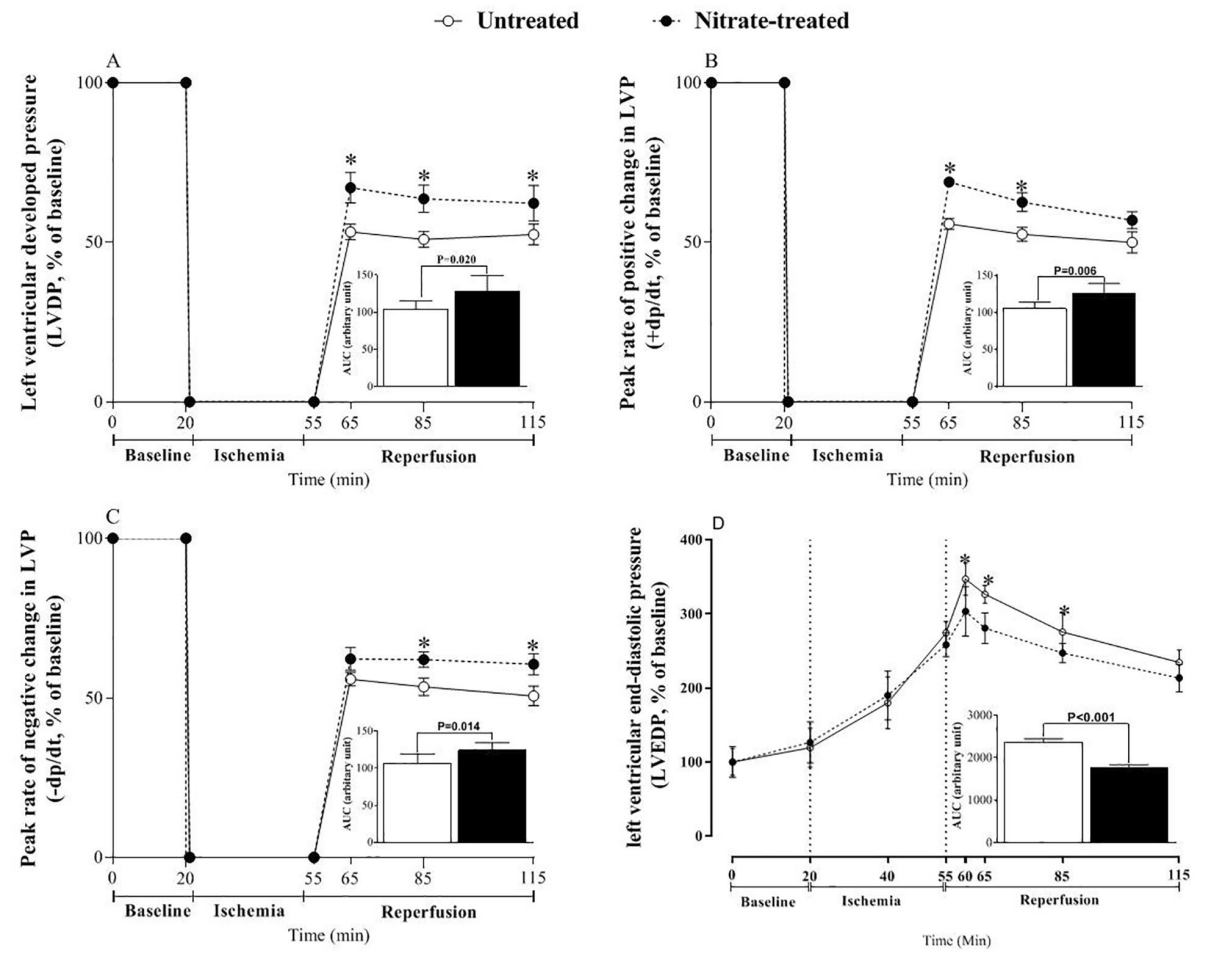
Resistance to myocardial ischemia-reperfusion injury (MIRI) following nitrate administration as measured by LVDP **(A)**, +dp/dt **(B)**,–dp/dt **(C)**, and LVEDP **(D)**. (n = 7/subgroup). Inset shows the area under the curves (AUC).

When subjected to ischemia, hearts from nitrate-treated rats showed better performance as displayed by increased recovery (decreased sensitivity to IR ) of LVDP (P = 0.020, Fig. 3A), +dp/dt (P = 0.006, Fig. 3B), and –dp/dt (P = 0.014, Fig. 3C) by 19%, 16%, and 14%, respectively. In addition, LVEDP was lower in nitrate-treated rats by 17% (P < 0.001, Fig. 3D).

**Fig. 4:**
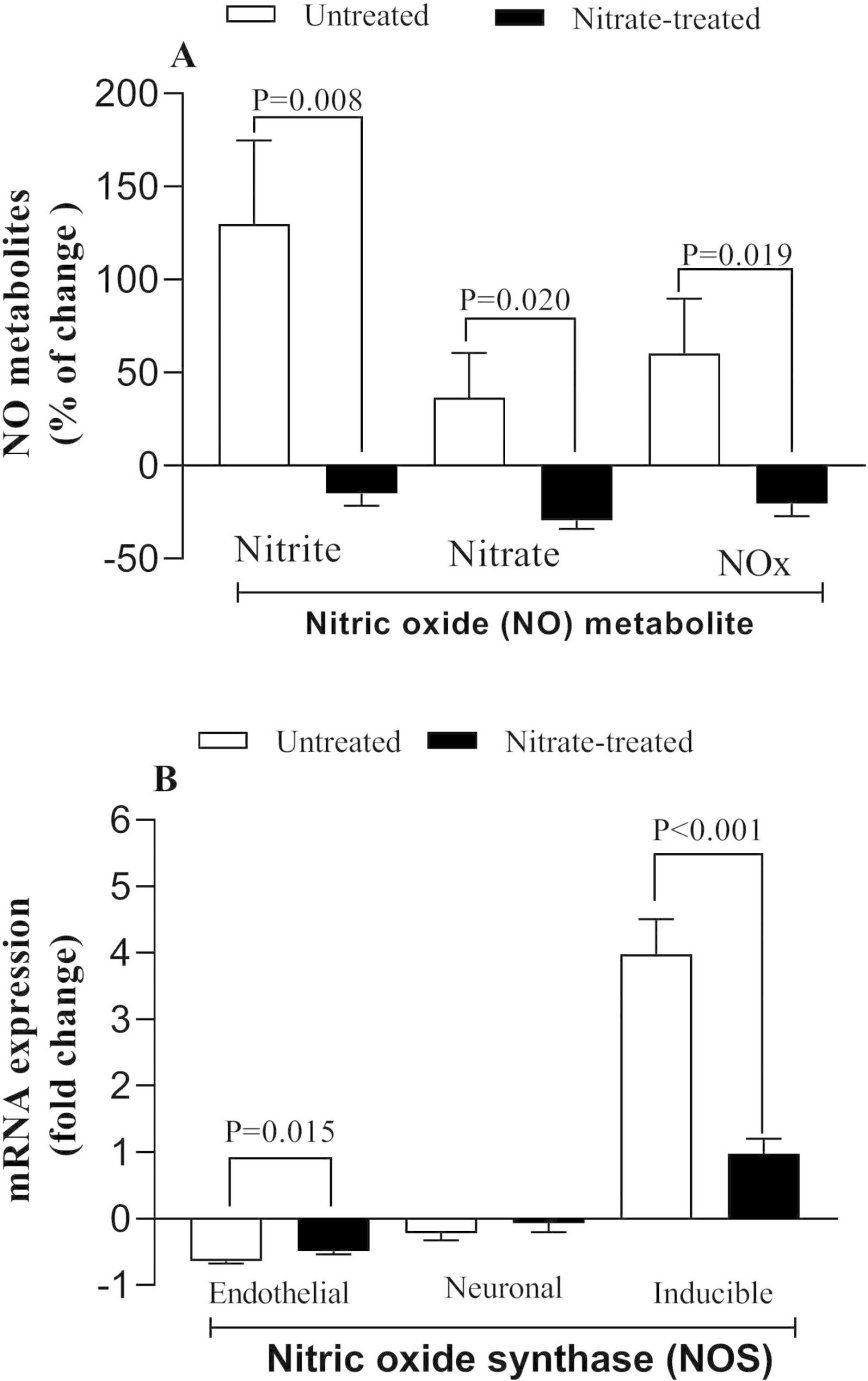
Heart levels of NO metabolites **(A)** and NOS mRNA expressions **(B)** following ischemia-reperfusion in untreated and nitrate-treated rats. n = 7/subgroup

When hearts of rats in untreated group were subjected to IR, heart nitrite, nitrate, and NOx changes significantly differed between untreated and nitrate-treated rats (Fig. 4A). In addition, eNOS and iNOS mRNA expression change were significantly different between untreated and nitrate-treated rats, whereas no changes in nNOS expression were observed between groups (Fig. 4B).

**Fig. 5:**
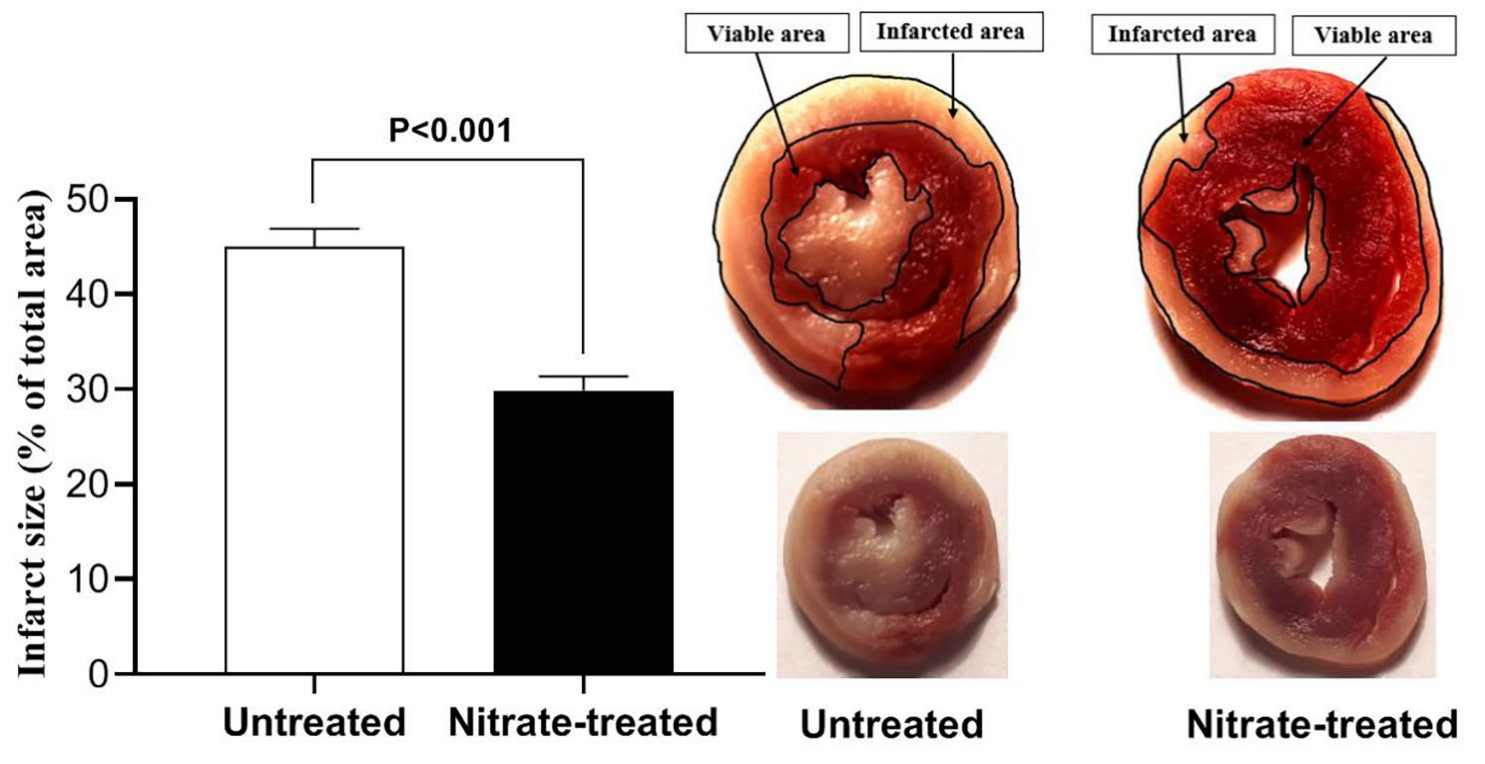
Myocardial infarct size following ischemia-reperfusion in untreated and nitrate-treated rats. (n = 7/subgroup)

Compared to untreated rats, hearts from nitrate-treated rats showed lower infarct size by 34% (P < 0.0001) when exposed to IR (Fig. 5).

## Discussion

The results of the current study showed cardioprotective effects of long-term nitrate preconditioning against MIRI as displayed by the increased recovery of LVDP, ±dp/dt, and decreased LVEDP and infarct size. These favorable effects of nitrate were related to increasing baseline eNOS expression before ischemia and to blunting decreased eNOS and increased iNOS expressions following subjection to IR.

In the present study, nitrate (100 mg/L for 9 months) decreased BW in female rats by 8.4%. This finding is in line with that obtained in healthy male rats (mean nitrate dose at 135 mg/L for 1.4–25 weeks) as reported in a meta-analysis [41]. The BW-lowering effect of nitrate in current work was not linked to food intake, as food intake was unaffected by the nitrate intervention. It has been reported that nitrate is converted to nitrite and then to NO, decreasing BW by increasing cyclic guanosine monophosphate [42] and browning of white adipose tissue [43].

In this study, hearts from nitrate-treated female rats showed more resistance to MIRI, as displayed by improved recovery of CFI and reduced infarct size. To the best of our awareness, no studies have addressed the effects of nitrate against MIRI in female rats at long-term. Previous in vitro studies have evaluated the cardioprotective effects of a single dose of nitrite applied before subjection to ischemia [44, 45] and before the onset of reperfusion [45, 46]. Moreover, protective effects of a single dose of nitrite against MIRI have been reported at dogs of either sex [44]. In addition, as summarized in Table 2, in vivo studies indicated that nitrite [24, 25] and nitrate [25, 26] administration for 7 days in mice or nitrate administration for 60 days [9] in male rats increase the resistance to MIRI. Our data extend this effect to 270 days in female rats.

**Table 2:** Summary of studies showing favorable in vivo effect of dietary nitrite/nitrate on myocardial ischemia-reperfusion injury in healthy rodents

Study	Animal	Sex	Intervention	Dose (mg/L in drinking water)	Duration (days)	Outcomes and mechanism
Bryan et al. -2007 [25]	Mice	Not reported	Nitrite	50	7	↓ infarct size by increasing serum and cardiac nitrite levels
Bryan et al. -2008 [24]	Mice	Not reported	Nitrite	50	7	↓infarct size by increasing cardiac nitric oxide (NO) metabolites levels
Bryan et al. -2007 [25]	Mice	Not reported	Nitrate	1000	7	↓infarct size by increasing plasma and heart nitrite
Salloum et al. -2015 [26]	Mice	Male	Nitrate	43*	7	↓infarct size and improved ventricular function
Jeddi et al. -2016 [9]	Rat	Male	Nitrate	100	60	Improved recovery of cardiac function by increasing cardiac endothelial NO synthase, decreasing cardiac inducible NO synthase, and decreasing oxidative stress

* Beetroot juice containing 0.7 mM nitrate

The positive effects of nitrate against MIRI observed in our study were related to increased cardiac eNOS expression (~ 200%) and increased circulating progesterone (~ 30%) before ischemia and blunting decreased eNOS and increased iNOS expressions after IR. The majority of the heart-associated NO production is derived from eNOS [11]. Under normal conditions, eNOS and nNOS produce about 80% and 20% of NOS-dependent NO production in the heart, with a negligible contribution from iNOS [11]. In line with our findings, myocardial-specific eNOS overexpression in male mice protects the heart against myocardial infarction (MI) and MIRI [14, 15], whereas eNOS knockout exacerbates MIRI [47]. We previously reported increased heart eNOS expression following nitrate intake for 60 days [9]. Moreover, an NO donor, nicorandil (3 mg/kg/day for 24 h), increases the heart expression of eNOS in male rats [48]. Our results indicated that preconditioning with nitrate in female rats increased heart nitrite (~ 130%) and NOx (~ 60%) concentrations, a result in line with that of Bryan et al., who reported increased heart nitrite levels following nitrite and nitrate intake for 7 days in mice [25], however, they did not specify animal sex.

Another mechanism underlining the favorable effect of nitrate against MIRI, observed in our study, is an increase in circulation progesterone levels. In parallel with this data, it has been stated that NO donors stimulate progesterone secretion in the cultured ovarian cells of rats [49]. Progesterone had defensive effects against IR injury in isolated hearts of female rats by decreasing infarct size [50], decreasing cardiomyocyte apoptosis [51], and decreasing fibrotic tissue [52]. Progesterone also increases the binding of specificity protein 1 and nuclear progesterone receptor to the eNOS promoter in endothelial cells [53], which subsequently enhances its activity and, eventually, the expression of eNOS. Additionally, progesterone attenuates the increase in iNOS expression in cerebral tissue [54].

Our results for the first time showed that preserved cardiac functions following subjection to ischemia in nitrate-treated female rats are associated with the blunted decrease in eNOS and increase in iNOS expressions. We previously reported a similar finding in male rats following 2-month nitrate administration [9]. During ischemia, NO in the heart is primarily produced by iNOS; thus, inhibition [55] or knockout [56] of iNOS protects, whereas iNOS overexpression exacerbates MIRI [57, 58]. iNOS-derived NO decreases eNOS expression in the rat heart tissue [59] and may produce peroxynitrite, thus contributing to MIRI [60]. mRNA levels of iNOS were higher in the cardiac tissue of male eNOS^−/−^ mice [61], and in male Sprague–Dawley rats, overexpression of iNOS led to decreases in eNOS expressions [59]. iNOS-derived NO decreased eNOS expression by increasing inflammatory biomarkers such as nuclear factor kappa B (NF-κB) and tumor necrosis factor-alpha (TNF-α) [62], leading to destabilized eNOS mRNA [16]. Reports strongly suggest that nitrate/nitrite-derived NO decreases levels of iNOS mRNA expression [63, 64] and serum IL-1β concentrations [65]. In cell lines, NO donors such as nicorandil [66], 1,1-diethyl-2-hydroxy-2-nitroso-hydrazine [67], and S-Nitroso-N-acetyl-d,l-penicillamine [67], decreased TNF-α levels [66] and increased TGF- β1 protein expression [67]. In endothelial cells, TGF- β1 increased eNOS mRNA expression [68], which was due to a direct interaction of the suppressor of mothers against decapentaplegic 2 (SMAD2, a downstream transcription factor in TGF- β1 signaling pathway) with the eNOS gene promoter [69]. Therefore, the stimulatory effects of nitrate on eNOS expression in our study may be related to decreasing iNOS expression and inflammation in the heart tissue.

Notably, protection against MIRI has also contributed to decreased SBP [70, 71] and increased HW/BW ratio [29]. However, nitrate administration in our study was not associated with changes in SBP, HR, or HW/BW ratio. In addition, our results showed that the baseline CFI were not significantly improved by long-term nitrate preconditioning, which is similar to our prior study following nitrate intervention in male rats [9], and other studies following single-dose administration of nitrite in male rats [45] and pigs [72].

As a strength, the dose of nitrate used in our current work (10.8 mg.kg^− 1^ per day) can be translated to an equivalent amount in humans (1.8 mg.kg^− 1^) [73], which is lower than the Acceptable Daily Intake of the nitrate ion (3.65 mg/kg). Furthermore, according to a systematic review, the average nitrate consumption in clinical studies varies from 1.28 to 2.14 mg.kg^− 1^ per day in diverse countries [74], and therefore the amount of nitrate used in current work is achievable via the intake of vegetables in humans [75]. This study has some limitations; first, we used the Langendorff apparatus to measure cardiac function that does not wholly reflect the in vivo response of the heart to IR injury [76]. However, this method is simple, provides reproducible results and can be used for pharmacological interventions [77]. Second, we did not assess the protein levels and activity of NOS enzymes; changes in mRNA expression are not always associated with protein expression and activity [78]. However, the association of mRNA expression of the NOS enzymes and their protein levels and activity has been reported; for instance, in the heart tissue, a significant correlation (r = 0.54) was seen between iNOS mRNA expression and protein activity [79]. In addition, a parallel increase in both eNOS mRNA (2.2 fold) and protein levels (62%) has been reported following nicorandil administration [80]. Also, a similar increase in eNOS, nNOS, and a decrease in iNOS mRNA and protein levels have been reported in the liver, soleus muscle, and adipose tissue of diabetic rats following 6-month of nitrate administration [81]. Third, we used only female rats; the National Institutes of Health (NIH) states that the use of both sexes in animal studies is essential [82], and the sex differences would be more tangible if we had done the experiments on both male and female rats instead of using only female rats. However, we provided a summary of previous works on the effect of nitrate/nitrite on myocardial tolerance against IR injury in male animals (Table 2).

## Conclusions

Long-term nitrate administration increased myocardial tolerance against IR injury in female rats. This protective effect was associated with increased heart eNOS expression and increased circulating progesterone before ischemia as well as to blunting ischemia-induced increased iNOS and decreased eNOS after IR. These findings imply that increased nitrate consumption, which is easily achievable via vegetable consumption [83], can reduce the risk of or attenuate the outcomes of cardiac events.

### Electronic supplementary material

Below is the link to the electronic supplementary material.

**Additional File Fig 1:** Randomization of rats using the random function of the Excel software, version 13.0.

## Data Availability

The datasets used and/or analyzed during the current study available from the corresponding author on reasonable request.
