# Supplementary material for: Long-term inorganic nitrate administration protects against myocardial ischemia-reperfusion injury in female rats
Source: BMC Cardiovasc Disord. 2023 Aug 21;23:411. doi: 10.1186/s12872-023-03425-2 (PMC10441752; doi:10.1186/s12872-023-03425-2)

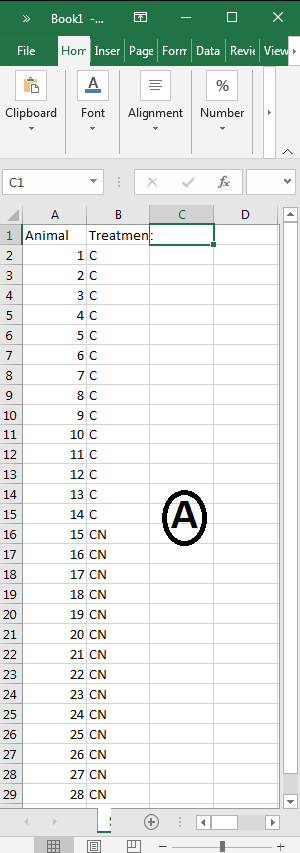

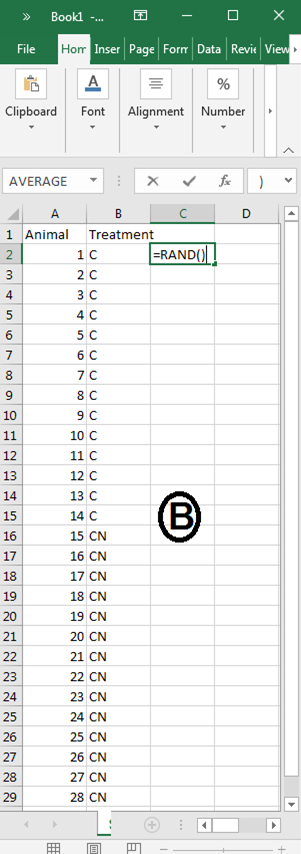

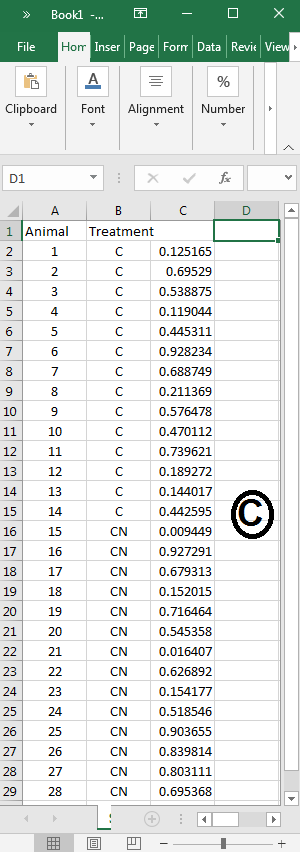

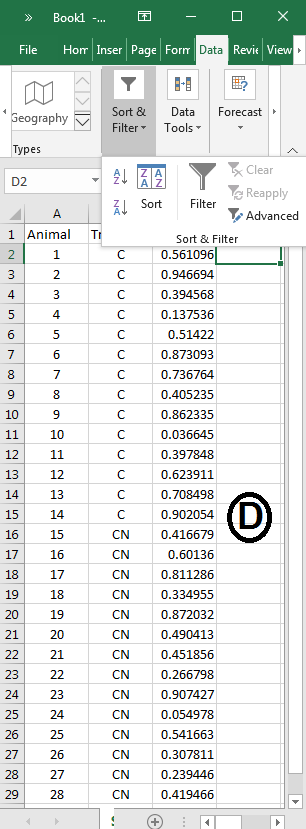

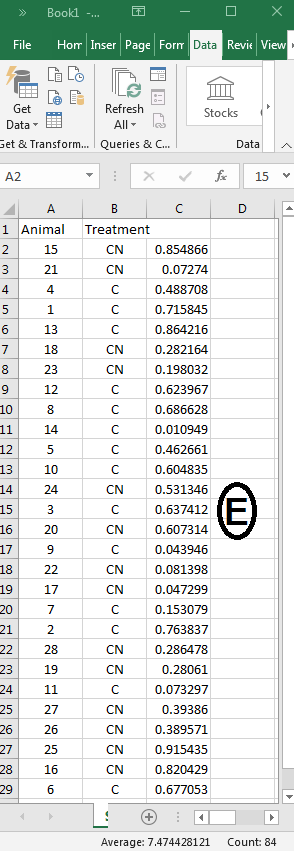


**Supplementary Figure 1.** Randomization of rats using the random function of the Excel software, version 13.0. In this study, 28 rats were randomized to 2 groups, including control, and control+nitrate (CN), 14 rats per group using the random function of the Excel software. To do this process: **(A)** Write animal ID in column A and study groups in column B in a nonrandom order, **(B)** Use Excel’s random number generator to create a random number between 0 and 1 in column C2. The formula required is: = RAND(), **(C)** Drag the formula in C2 down to fill the remaining cells C3 to C41. This creates a list of 28 random numbers between 0 and 1. **(D)** Highlight the group column (column B) and the random numbers column (column C). **(E)** Use Excel's Data → Sort command to sort column B by column C. We have now randomized the group labels of the design to the rats (Figure 1.E).


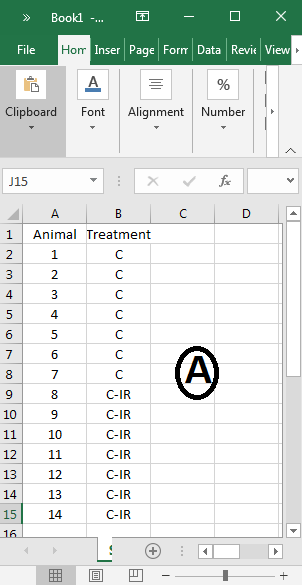

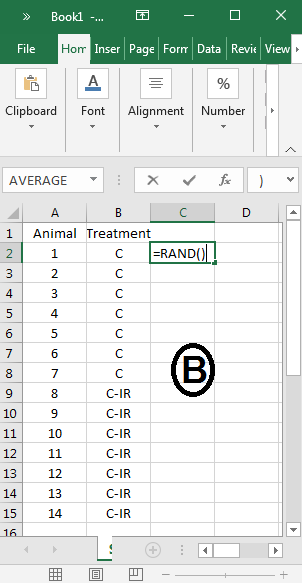

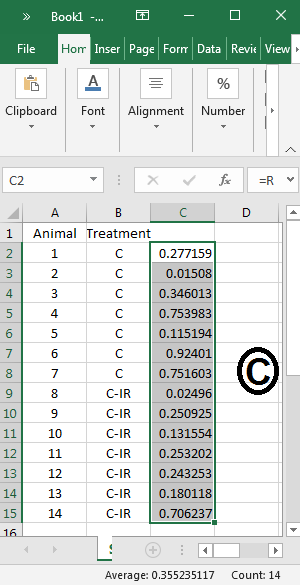


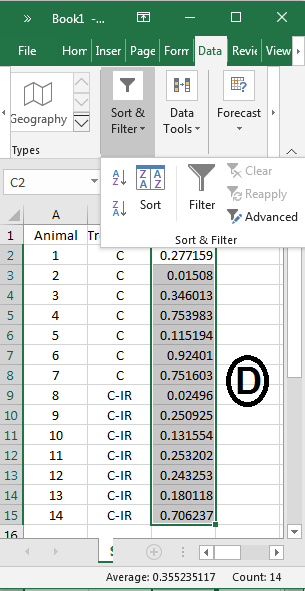

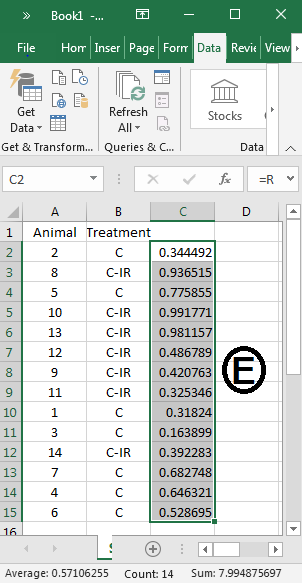


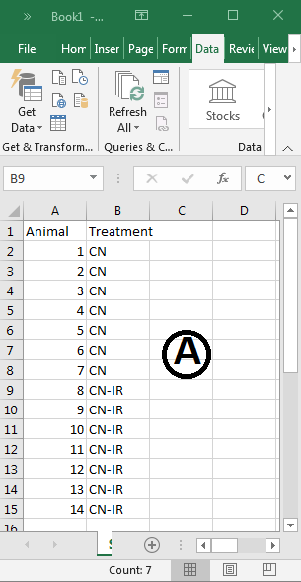

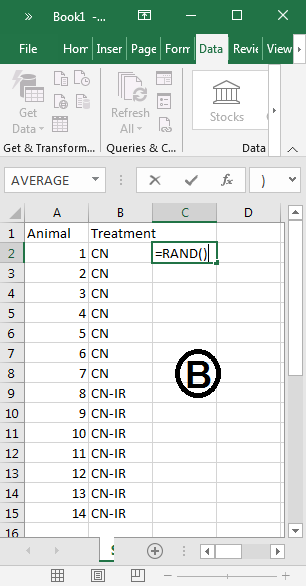

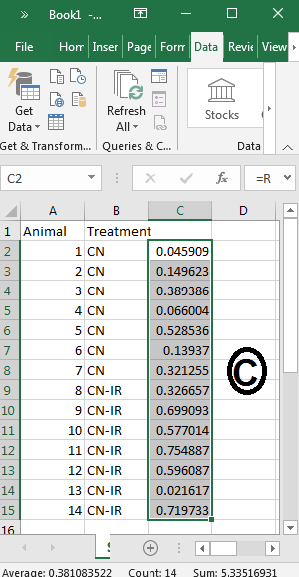

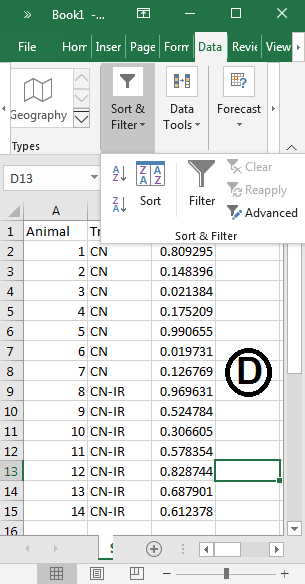

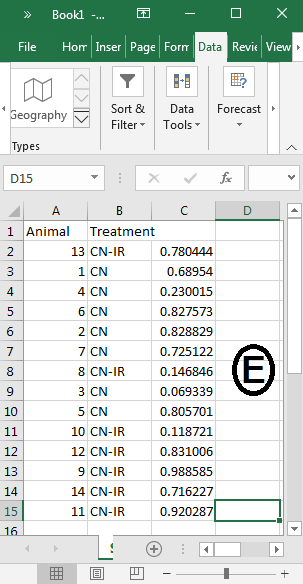

Supplement: Supplementary file 1 — Additional File Fig 1: Randomization of rats using the random function of the Excel software, version 13.0. [file 12872_2023_3425_MOESM1_ESM.docx]
